# ﻿*Oreocharisqianyuensis*, a new species of Gesneriaceae from Southwest, China based on morphological and molecular evidence

**DOI:** 10.3897/phytokeys.213.84349

**Published:** 2022-11-15

**Authors:** Jia-Wen Yang, Xin-Mei Qin, Jian Xu, Cong-Rui Li, Qi-Fei Ren, Mao-Qin Yuan, Qiang Zhang, Si-Rong Yi, Lei Cai

**Affiliations:** 1 Guizhou Botanical Garden, Guiyang 550004, Guizhou, China; 2 Guangxi Key Laboratory of Plant Conservation and Restoration Ecology in Karst Terrain, Guangxi Institute of Botany, Guangxi Zhuang Autonomous Region and the Chinese Academy of Sciences, Guilin 541006, China; 3 Guizhou Academy of Forestry, Guiyang 550000, Guizhou, China; 4 Chongqing Three Gorges Medical College, Chongqing 404120, China; 5 Yunnan Key Laboratory for Integrative Conservation of Plant Species with Extremely Small Populations, Kunming Institute of Botany, Chinese Academy of Sciences, Kunming 650201, Yunnan, China; 6 Key Laboratory for Plant Diversity and Biogeography of East Asia, Kunming Institute of Botany, Chinese Academy of Sciences, Kunming 650201, Yunnan, China

**Keywords:** Flora of China, Morphology, *Oreocharis*, Phylotranscriptomics

## Abstract

*Oreocharisqianyuensis*, a new species of Gesneriaceae from Southwest, China, is described and illustrated based on morphological comparisons and molecular phylogenetic analyses. Phylotranscriptomic analyses of the new species in the context of a comprehensive phylogeny with dense sampling of 88% (111/126) of all species of the genus indicated that the new species was most closely-related to *O.fargesii*. The new species is morphologically similar to *O.fargesii* and *O.nanchuanica* in the shape, color and structure of flowers and the number of stamens, but differs in the leaf blade shape, margin and the indumentum characters of the inflorescence. Its morphological relationship with similar species is discussed, the detailed descriptions, colour photographs, distribution, as well as the IUCN threatened status based on the IUCN Red List Categories and Criteria are also provided.

## ﻿Introduction

Möller et al. (2011) redefined *Oreocharis* s.l. (Gesneriaceae) and recognized 102 species. In the following decade of research, some taxa (e.g., *Ancylostemondimorphosepalus* W.H. Chen & Y.M. Shui, *Beccarindabaolianis* Q.W. Lin, *Boeicaguileana* B.L. Burtt, *Briggsiaacutiloba* K.Y. Pan, *B.muscicola* (Diels) Craib, *Tremacronhongheense* W.H. Chen & Y.M. Shui) were incorporated into the genus (Burtt 1977; Pan 1988; Chen et al. 2012, 2014; Middleton et al. 2013; Möller et al. 2014; Cai et al. 2015; Möller 2015; Lin 2016; Yang et al. 2021; *Bourneasinensis* Oliv. and *B.leiophylla* (W.T. Wang) W.T. Wang & K.Y. Pan were removed from the genus based on molecular and palynological evidence (Chen et al. 2020), together with the publication of some dozens of new species (e.g., Wei et al. 2016; Cai et al. 2017, 2020; Guo et al. 2018; Pan et al. 2019; Yang and Shi 2021; Le et al. 2022), *Oreocharis* s.l. hitherto comprises ca. 160 species, mainly distributed in South and Southwest China (150 species), with several species occurring in North Vietnam (ten species with eight endemic), Myanmar (two species), Bhutan (one species), India (one species), Japan (one endemic species) and Thailand (one endemic species) (Wang et al. 1990, 1998; Li and Wang 2005; Cai et al. 2020; Wen et al. 2021). *Oreocharis* shows extremely diverse floral charateristics, particularly regarding the corolla shape ranging from narrowly or widely tubular, campanulate, urceolate, to flat; symmetry from zygomorphic to actinomorphic; color from white, red, yellow, pink to purple (Jin et al. 2021). A recent extensive study based on transcriptomic data of 88% (111/126) of all species of the genus revealed the spatiotemporal diversification and the possible driving forces (Kong et al. 2022). This study provided a robust phylogenetic hypothesis of the relationships of most species and acts as firm basis for further studies such as species identification and delimitation.

In 2019, during a field investigation in Kaili City, Guizhou, China, an anomalous plant of Gesneriaceae with few flowers caught the authors’ attention, and in July 2020, it was recollected at flowering time. Coincidentally, similar specimens were collected by Si-Rong Yi from Pengshui County, Chongqing, China in 2021. We identified it as a member of the previously recognised genus of *Isometrum* Craib (Pan 1986; Wang et al. 1990; Li and Wang 2005), which now belongs to the genus *Oreocharis* s.l. based on the flower and fruit characteristics, such as: four stamens coherent in pairs, anther thecae not confluent, capsule dehiscent on one side (Wang et al. 1990, 1998; Li and Wang 2005). After examination of the specimens stored in the related herbaria (E, HITBC, IBK, HN, K, KUN, P, PE and VMN) including digital specimens online, such as Chinese Virtual Herbarium (http://www.cvh.ac.cn/) in China and Global Plants on JSTOR (https://plants.jstor.org/), and consulting the related taxonomic publications of *Oreocharis* from the adjacent regions (Wang et al. 1990, 1998; Li and Wang 2005; Li and Li 2015; Guo et al. 2018; Cai et al. 2019; Fu et al. 2019a, b), we could not match the species to any previously published one of this genus. Then we carried out transcriptome sequencing using the leaves from two individuals of Guizhou Population and two individuals of Chongqing Population respectively, and added them to the recently published large data set of orthologous nuclear genes screened from the transcriptomic data of 111 *Oreocharis* species to reconstruct the phylogeny of the genus including the suspected new species (Kong et al. 2022). The results suggested that the plants were nested within *Oreocharis* and the analysed material was phylogenetically distinct from other species. Here, *Oreocharisqianyuensis* Lei Cai, J.W.Yang & Q.Zhang is described and illustrated based on the morphological comparisons and molecular phylogenetic analyses.

## ﻿Materials and methods

We measured and recorded the morphological characters at least from more than ten mature individuals at flowering and fruiting from Guizhou and Chongqing populations. In addition, four relatively young leaves from each of the four individuals (two from Guizhou population and two from Chongqing population) were collected and sent to Novogene Technology Co., Ltd. for transcriptome sequencing. After filtering the low-quality reads, the remaining clean reads were used for denovo assembling with the package Trinity v2.11.0 (Grabherr et al. 2011). Referring to the published data consisting of 574 orthologous genes and including 111 *Oreocharis* species (Kong et al. 2022), we extracted the corresponding orthologous genes and added them to the data set for phylogenetic reconstruction. Phylogenetic tree was inferred based on the data set of the concatenated genes using maximum likelihood (ML) in RAxML v8.0.X (Stamatakis 2014) with parallel computation employing 100 threads on a server (ThinkSystem SR860). The parameters were set as GTR substitution model and a random starting tree with all others left as default. 100 bootstrap replicates were used to assess the robustness of the branches in the ML tree.

### ﻿Data availability statement

The transcriptome data of four individuals in this study are openly available from NCBI: https://www.ncbi.nlm.nih.gov/sra/PRJNA813939 (the two individuals from Chongqing population) and https://www.ncbi.nlm.nih.gov/sra/PRJNA861104 (the two individuals from Guizhou population).

## ﻿Results

**Figure 1.**
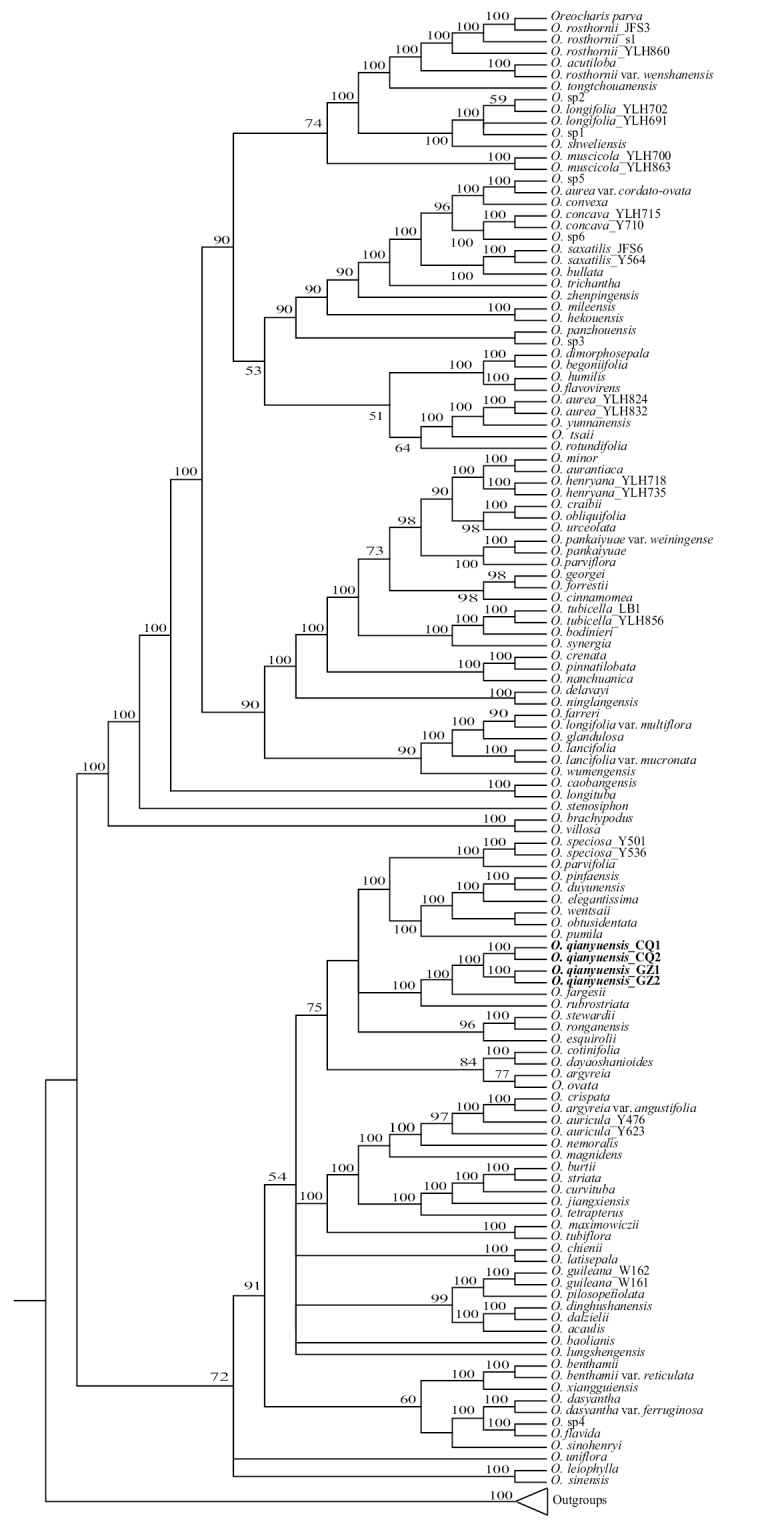
A maximum likelihood (ML) phylogeny of *Oreocharis* based on the concatenated data set of 573 loci with bootstrap support values (> 50%) shown below or above the branches around the corresponding nodes.

For the 574 target nuclear orthologous genes, 566, 566, 561, 567 nuclear orthologous genes were screened out from each of the four transcriptomes (*Oreocharis qianyuensis_*CQ1, *O. qianyuensis_*CQ2, *O. qianyuensis_*GZ1, *O. qianyuensis_*GZ2), respectively, and one gene failed to be obtained from any of the four individuals. Hence 573 genes were included and the concatenated matrix had a length of 839193 bp. The matrix contained 376988 variable sites and 203260 parsimony informative sites, with an overall average GC content of 44.39%. The phylogenetic analyses using ML showed that the four individuals of the new species were clustered together and they in turn were grouped with *O.fargesii* (Franch.) Mich. Möller & A. Weber with full support (BS=100%), followed by *O.rubrostriata* F. Wen & L.E. Yang (BS=100%) in a lineage in *Oreocharis* (Fig. 1).

### ﻿Taxonomic treatment

#### ﻿ Oreocharis qianyuensis

Taxon classificationPlantaePasseriformesParamythiidae

Lei Cai, J.W.Yang & Q.Zhang sp. nov.

03FB2CE5-E2EB-5DDE-B805-AC031BE70ADE

urn:lsid:ipni.org:names:77308124-1

Figs 2 , 3 , 4 , 5

##### Diagnosis.

The new species is morphologically most similar to *Oreocharisfargesii* (Franch.) Mich. Möller & A. Weber and *O.nanchuanica* (K.Y. Pan & Z.Y. Liu) Mich. Möller & A. Weber in the color, shape and structure of flowers, but differs from the latter two in its inconspicuously petiolate, obovate to flabellate leaf blade with adaxially surface sparsely rust-brown pubescent to glabrescent, abaxially densely rust-brown villous, apex rounded, base extending downward into a wing shape, margin crenate, and the peduncle and pedicel densely glandular pubescent. A comparison of morphological differences between the related species is provided in Table 1.

**Table 1.** Morphological comparison among *Oreocharisqianyuensis* sp. nov., *O.fargesii* and *O.nanchuanica*.

Characters	*O.qianyuensis*	*O.fargesii*	*O.nanchuanica*
**Leaf blade**	obovate to flabellate, adaxially sparsely rust-brown pubescent to glabrescent	obovate to narrowly oblong, sometimes oblique, adaxially appressed puberulent-strigillose	ovate, adaxially appressed gray puberulent
**apex**	rounded, obtuse to nearly truncate	rounded to obtuse	acute
**margin**	crenate	serrate near apex	serrate
**base**	extends downward forming a wing	cuneate	cordate
**Peduncle indumentum**	rust-brown villous and densely glandular pubescent	rust-brown villous, glabrescent	brown puberulent and glandular puberulent
**Petiole and indumentum**	almost invisible	to 1.5 cm long, rust-brown villous	to 8.3 cm long, brown pubescent
**Bract indumentum**	rust-brown villous	rust-brown villous	brown puberulent
**Pedicel indumentum**	rust-brown villous and glandular pubescent	rust-brown villous, glabrescent	brown puberulent and glandular puberulent
**Calyx lobes**	lanceolate triangle, margin denticulate	lanceolate to triangular, margin entire	lanceolate, margin entire
**Corolla tube**	campanulate, not constricted at throat	campanulate, not constricted at throat	urceolate, constricted at throat
**Staminode**	ca. 0.5 mm long	ca. 2 mm long	ca. 0.7 mm long

##### Type.

China, Guizhou Province: Kaili City, Dafengdong Town, Shuangjiangkou Village, Taiyanghe, 26°42'30"N, 107°49'32"E, elev. ca. 845 m, on the surfaces of rocks under the thicket, in flowering, 15 July 2020, Jia-Wen Yang et al. CL2020247 (Holotype: KUN!; Isotypes: KUN!, P!).

##### Description.

Perennial herb, rhizome short. Leaves 4–7, basal; without petiole or extremely inconspicuous petiole, leaf blade obovate to flabellate, 3.0–12 × 2.0–8.5 cm, adaxially sparsely rust-brown pubescent to glabrescent, abaxially rust-brown villous, densely along veins, lateral veins 3–6 on each side of midrib, apex rounded, obtuse to nearly truncate, base extends downward into wing shape, basally enclosed with dense and long rust-brown villous tuft, margin crenate. Cymes axillary 2–5, 2–12-flowered per inflorescence; peduncle 4.5–14 cm long, cover with rust-brown villous and densely glandular pubescent, basally enclosed with dense and long rust-brown villous tuft; bracts 2, linear triangle to lanceolate, margin entire, 3.0–4.5 × 1.6–2.0 mm, outside rust-brown villous; pedicel 2.0–5.0 cm long, densely glandular pubescent. Calyx 5-parted to base, lobes equal, lanceolate triangle, 3.0–5.0 × 1.0–1.4 mm, margin denticulate, outside rust-brown villous, inside glabrous. Corolla brownish red to dark purple, 7–12 mm long, outside and inside glabrous, tube campanulate, 5–8 mm long, 4.5–5.5 mm in diameter at the widest position; limb 2-lipped; adaxial lip 2-lobed from the middle, lobes semiorbicular, 3.5–4.5 × 2.5–3 mm, abaxial lip 3-lobed to base, lobes semiorbicular, 4–4.5 × 3–3.5 mm. Stamens 4, 4.5–6 mm long, adnate to corolla 2–2.5 mm from base; filaments linear, glabrous; anthers broadly ovate, 2-loculed, coherent in pairs, connective glabrous; staminode 1,ca. 0.5 mm long, inserted ca. 1 mm from base. Disc 1–1.5 mm high, yellow, margin undulate. Pistil 5–8 mm long; ovary long cylindrical, glabrous, 3.0–4.5 mm long; style ca. 2.0–3.5 mm long, glabrous; stigma orbicular, emarginate in the middle, slight bilobed when dry. Capsule linear, glabrous, 2.0–2.8 cm long, 2–3 mm in diameter, dehiscing predominantly on one side.

**Figure 2.**
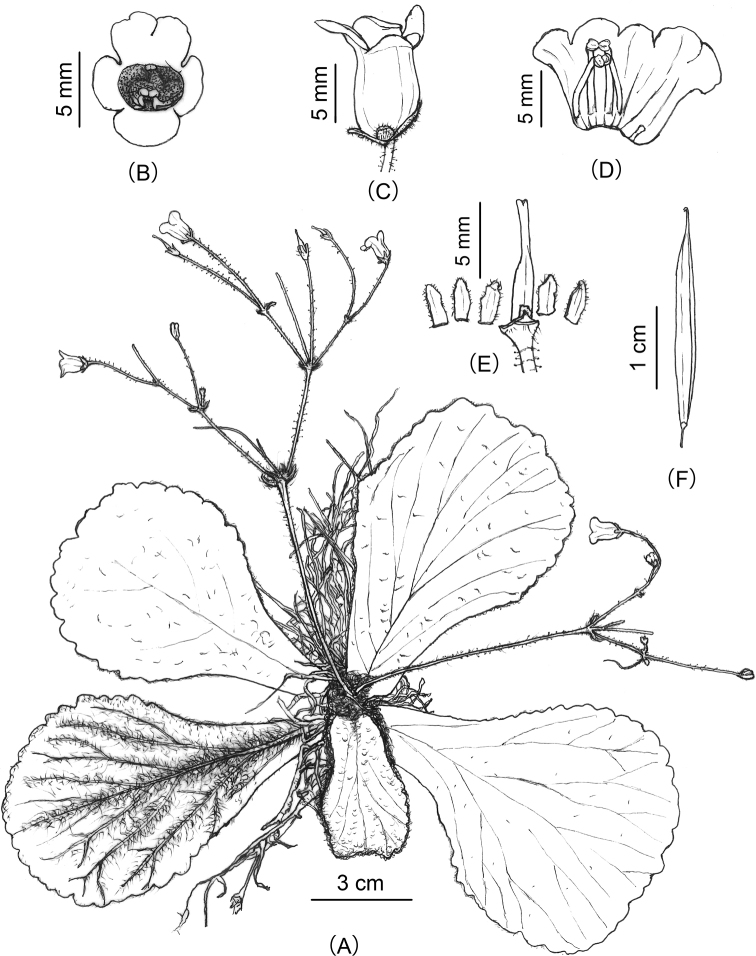
*Oreocharisqianyuensis* sp. nov. **A** habit **B** front view of a flower **C** side view of a flower **D** opened corolla showing stamens and staminode **E** pistil with disc and calyx **F** old fruit. Drawn by Xuan-Lin Zhu.

**Figure 3.**
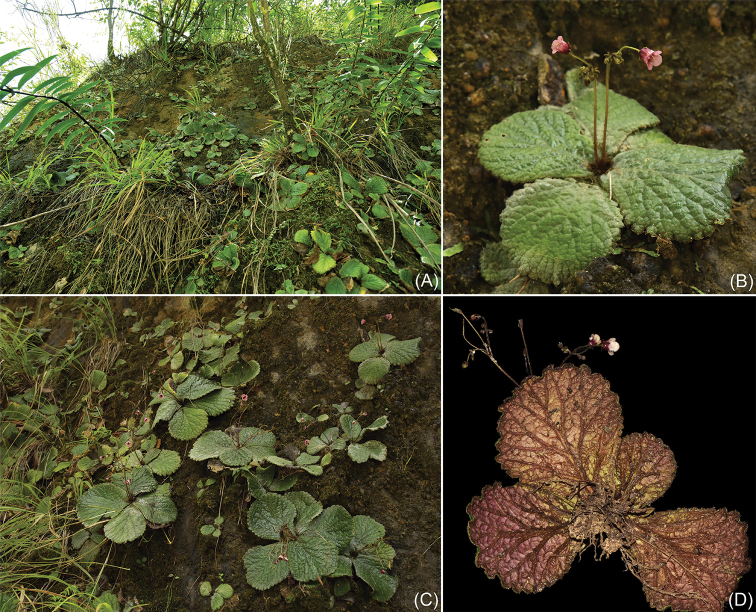
*Oreocharisqianyuensis* sp. nov. (Population in Guizhou) **A, C** habitat **B, D** plants with flowers.

**Figure 4.**
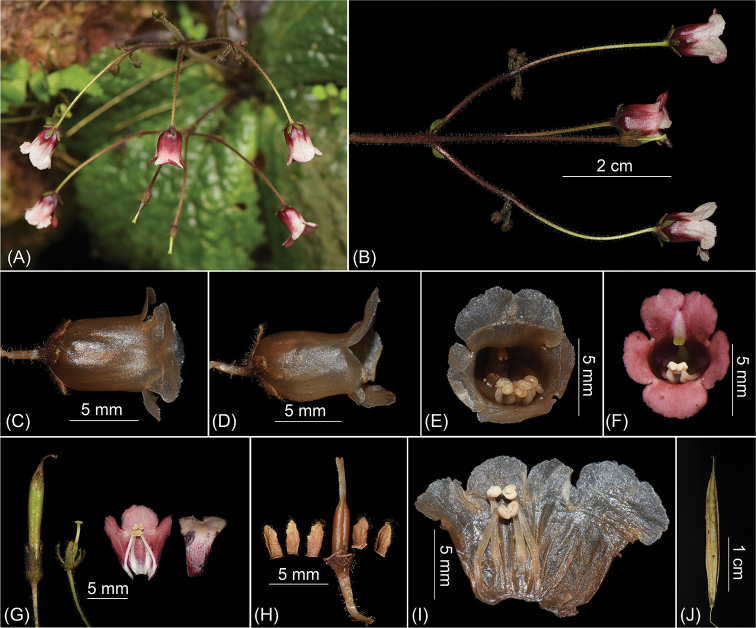
*Oreocharisqianyuensis* sp. nov. (Population in Guizhou) **A, B** inflorescence **C** top view of flower **D** side view of flower **E, F** front view of flowers **G** young fruit, pistil with disc and calyx, opened corolla showing stamens and staminode **H** pistil with disc and calyx **I** opened corolla showing stamens and staminode **J** old fruit.

**Figure 5.**
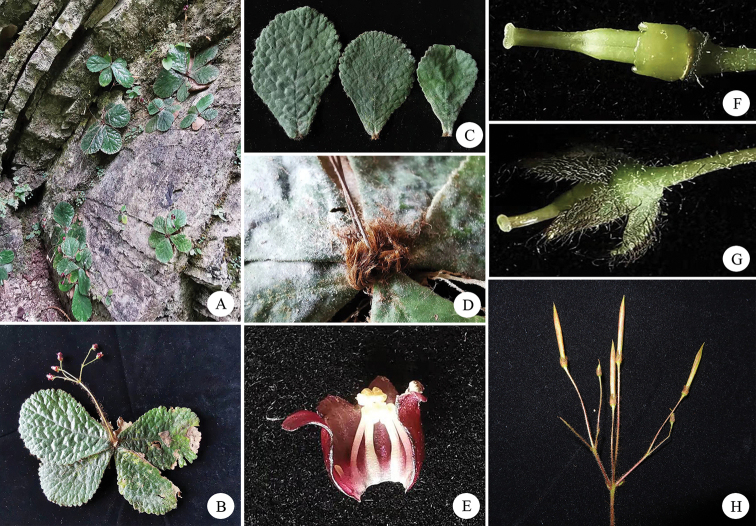
*Oreocharisqianyuensis* sp. nov. (Population in Chongqing) **A** habit **B** plant with flowers **C** leaves **D** pilose tuft **E** opened corolla with stamens and staminode **F** pistil and disc **G** calyx with pistil **H** infructescence.

##### Phenology.

Flowering from July to August in Guizhou and from September to October in Chongqing; time of fruiting unknown.

##### Etymology.

The specific epithet ‘*qianyuensis*’ refers to the known distribution at the time of publication in Guizhou and Chongqing in China. Qian is an alternative name for Guizhou and Yu is an alternative name for Chongqing.

##### Vernacular name.

The Chinese name of the new species is “Qian Yu Ma Ling Ju Tai” (黔渝马铃苣苔). The first two characters mean this species is distributed in Guizhou and Chongqing, and the last four characters represent the Chinese name of the genus *Oreocharis*.

##### Distribution and conservation status.

*Oreocharisqianyuensis* was observed to grow on the surfaces of rocks under forest in karst region in Kaili City, Guizhou, and on limestone rock surface or crevices under deciduous forests in Pengshui County, Chongqing. The species is currently known from one population of ca. 2000 individuals within 5500 m^2^ (AOO) in Guizhou and one population of ca. 300 individuals within 1000 m^2^ (AOO) in Chongqing. Since no special surveys were carried out for its distribution, and the threat is that the population is close to roadside in Guizhou and possible continuous drought in Chongqing, so it is very likely to be damaged or excavated, so this species was provisionally considered to be Endangered [EN B2ab(iii)] in terms of IUCN Red List categories and criteria (IUCN 2022).

##### Specimens examined.

China. Chongqing: Pengshui County, Hanjia Town, on rock walls, 29°1'90.94"N, 108°13'23.16"E, elev. 290 m, 4 October 2021, Si-Rong Yi YSR9297 (Paratypes: IBK!).

### ﻿Taxonomic affinities

The molecular evidence (phylogenetic tree) clearly supports that this new species belongs to the genus *Oreocharis* s.l. (Fig. 1). Our phylogenetic results presented here are congruent with the results presented by Chen et al. (2020), and here, we choose to support the decision of removing the genus *Bournea* from the enlarged *Oreocharis*. Although the latest conclusions from the phylogenetic study of *Oreocharis* s.l. by Lv et al. (2022) are different, which may be related to the data these authors used providing a different view of early and rapid evolutionary radiation of the *Oreocharis*. Further work is needed to clarify these incongruent results. Another important aspect for us is to classify this species into this genus based on some characteristics of its flowers and capsules, such as: four stamens coherent in pairs, anther thecae not confluent, capsule dehiscent on one side (Wang et al. 1990, 1998; Li and Wang 2005). *O.qianyuensis*, *O.fargesii* and *O.nanchuanica* have characters shared with the previously recognized genus *Isometrum* Craib based on the anthers attached in pairs, corolla purple, tube campanulate and not swollen (Pan 1986; Wang et al. 1990, 1998; Pan and Liu 1995; Li and Wang 2005). *O.qianyuensis*, *O.fargesii* and *O.nanchuanica* were listed as members of Sect. Pachysiphon K.Y. Pan and can be distinguished from the other *Isometrum* species by their corolla tubes short and thick (e.g., 6–12 mm long, 5–9 mm in the diameter, the length is 1.2–1.5 times the width), outside glabrous (Pan 1986; Wang et al. 1990, 1998; Li and Wang 2005). *O.qianyuensis* morphologically resembles *O.fargesii* and *O.nanchuanica* in the purple flowers, 5-parted to the base of calyx, campanulate corolla and coherent in pairs of anthers, however, *O.qianyuensis* can be easily differentiated from them by the shape, margin, apex and base of leaf blade shape, indumentum characters of the inflorescence. Detailed diagnostic characters of the new species are listed and compared with other morphologically similar species in Table 1.

## Supplementary Material

XML Treatment for Oreocharis qianyuensis
